# Youth, work, and equity: rethinking decent work through gender lens

**DOI:** 10.3389/fsoc.2025.1674310

**Published:** 2025-11-28

**Authors:** Cláudia Andrade, Paula C. Neves, Inês Bessa

**Affiliations:** 1 Escola Superior de Educação do Politécnico de Coimbra, Coimbra, Portugal; 2 InED—Centro de Investigação e Inovação em Educação, Politécnico do Porto, Porto, Portugal; 3 Centro de Psicologia da Universidade do Porto, Porto, Portugal

**Keywords:** youth, decent work, gender, equity, work

## Abstract

**Introduction:**

Decent work and gender equality are critical for fostering sustainable economic and social development. Even though the last three decades have seen several positive advancements in gender equality, disparities still exist in several ways, with research indicating that young women face more obstacles to employment and professional advancement than their male counterparts.

**Methods:**

An on-line cross-sectional survey was conducted with a sample of 190 Portuguese young adults who work to examine key factors of decent work such fundamental principles and values at work, adequate working time and workload, fulfilling and productive work, meaningful remuneration for the exercise of citizenship, social protection, opportunities and health and safety.

**Results:**

The analysis reveals gender differences in most of the dimensions of decent work, suggesting that gender plays a significant role in shaping these perceptions. Findings also suggested differential effects of having a team leader position and work schedules for male and female young workers.

**Discussion:**

Understanding these gendered perceptions is crucial for policymakers and employers aiming to promote gender equality and decent work for all. By addressing the specific perceptions of decent work both men and women using the gender equity lens can raise awareness about the barriers that act against equitable work environments, enabling to both men and women to create a positive future for themselves, their families, and their communities.

## 1 Introduction

The contemporary labor market is marked by growing complexity, flexibility, informal and precarious work, diminishing worker rights, and persistent disparities across regions and demographic groups (International Labour Organization, 2020). Decent work, as conceptualized by the International Labour Organization (ILO), represents employment that is productive, delivers a fair income, ensures workplace security, offers social protection for families, provides prospects for personal development, and promotes social integration, highlighting the importance of equal opportunities and treatment for all workers, alongside with freedom of expression and participation in decision-making processes (International Labour Organization, 2022).

The Psychology of Working Theory (PWT) by Blustein et al. (2016) offers a comprehensive framework for understanding how work influences individual well-being. This theory expands traditional vocational models by emphasizing access to decent work, the role of social and economic constraints, and the importance of fulfilling psychological needs such as autonomy, competence, and relatedness (Blustein et al., 2016). According to PWT, work is not only a means of financial survival but also a crucial context for identity formation, social connection, and psychological fulfillment (Blustein et al., 2016; Duffy et al., 2016). The theory highlights that barriers like discrimination, poverty, and limited education often restrict individuals’ access to meaningful employment, impacting mental health and life satisfaction. By incorporating constructs like critical consciousness and proactive engagement, PWT underscores how individuals can navigate structural inequalities. Recent empirical studies support the importance of PWT theory showing that decent work is positively linked to psychological well-being and life meaning (Kim et al., 2021) with PWT been a particularly influential framework to guide interventions that promote equity and career development for certain populations (Duffy et al., 2016). Following this line of research Ferraro et al. (2018) identified seven essential dimensions of decent work, including fundamental principles and values at work, adequate working time and workload, productive and rewarding work, meaningful remuneration for the exercise of citizenship, social protection, opportunities and health and safety, acknowledging cultural differences and the evolving nature of work.

This study aims to examine gender differences in young Portuguese workers’ perceptions of decent work, drawing on the Psychology of Working Theory (PWT) framework and the Decent Work Questionnaire dimensions (Ferraro et al., 2018). Specifically, the study investigates how factors such as leadership roles and work schedules may moderate these perceptions, with the broader goal of identifying structural barriers that hinder gender equity in employment.

### 1.1 Decent work and youth

The concept of *youth* is socially and historically constructed, encompassing a transitional life stage between dependence and autonomy, often marked the entry into the labor market emphasizing that youth cannot be defined solely by age but must be understood in relation to sociocultural, economic, and institutional contexts that shape opportunities and constraints (Furlong, 2013).

Studies conducted by the International Labour Organization (2020) indicate that young adults, regardless of whether they live in developed, developing, or underdeveloped nations, struggle to secure decent work. Similarly, in Portugal, national observatories have identified structural difficulties in the transition from school to stable employment (Youth Employment Observatory, 2024). Several barriers contribute to this challenge, including early school dropout, unstable family environments, societal biases against youth, limited access to public employment programs, weak social support, and the increasing prevalence of insecure jobs (Blustein et al., 2016; International Labour Organization, 2020). These conditions restrict access to stable, well-paying jobs, making it harder for young adults to establish a secure career. Consequently, many young adults experience career instability, moving between precarious jobs (OECD, 2016). In Portugal, the transition from education to employment and the initial years in the labor market present significant challenges for Portuguese youth. These challenges encompass high unemployment rates, skill mismatches, precarious employment conditions, and a pronounced trend of emigration among young professionals (Tavares et al., 2021). This precariousness is often characterized by temporary contracts, limited job security, and inadequate social protection, which can hinder long-term career development and financial stability. Considering the psychology of work theory framework this situation threatens their identity formation, hinders self-determination, and negatively impacts social relationships (Blustein et al., 2016; Duffy et al., 2016).

### 1.2 Gender differences in perceptions of decent work in young adults

In Portugal, sociocultural backgrounds play a decisive role in shaping gender stereotypes, educational pathways, and labor-market participation. Although the country has made substantial progress toward gender equality through legislative reforms and rising female educational attainment, cultural norms and gendered expectations continue to influence individual choices and opportunities. In the labor market, gender disparities remain pronounced (Santos et al., 2024). Women in Portugal face persistent wage gaps, occupational segregation, and difficulties reconciling work and family life, particularly in contexts where long working hours and limited institutional childcare prevail (Matias et al., 2011; Santos et al., 2024). Gender differences in decent work persist globally, reflecting inequalities in access, conditions, and treatment within the labor market with women disproportionately represented in informal, low-paid, and vulnerable employment, often lacking social protections and career advancement opportunities (International Labour Organization, 2022). Despite progress in education and workforce participation, structural barriers such as discriminatory hiring practices, gendered occupational segregation, and unequal caregiving responsibilities continue to limit women’s access to decent work, with men having higher opportunities to have leadership roles and better job security (International Labour Organization, 2022). In Portugal, gender differences in decent work remain evident despite legislative efforts to promote equality. The gender pay gap persists, with women earning approximately 13% less than men on average, even in comparable roles (Eurostat, 2025). Women are also overrepresented in lower-paid sectors such as education, health, and social services and remain underrepresented in managerial and decision-making roles and are more likely to occupy part-time or irregular jobs with less favorable working hours (Ferreira and Coelho, 2022; OECD, 2021). These inequalities reflect persistent structural and cultural barriers that hinder gender equity in employment. Among young adults, perceptions of what constitutes decent work are shaped by gendered expectations and socio-cultural contexts (Tavares et al., 2021). Research suggests that young men and women often prioritize different aspects of work due to socialization and labor market realities (Hirschi, 2012). Young women are more likely to value job security, work-life balance, and supportive workplace environments, often due to expectations surrounding future caregiving roles (Duffy et al., 2016; International Labour Organization, 2022), while young men may place greater emphasis on income potential, autonomy, and advancement opportunities, reflecting traditional notions of male breadwinning (Hirschi, 2012). These structural inequalities and gendered perceptions can influence career choices, occupational segregation, and even long-term job satisfaction. Women face higher risks of job precarity, wage gaps, and more limited career prospects, which may make them more sensitive to fairness and inclusivity when assessing job quality (Tavares et al., 2021). Understanding these gender differences is crucial for policymakers and educators aiming to promote equitable labor participation among youth. Overall, gender theory offers a critical lens for understanding how structural inequalities and social expectations shape access to decent work by highlighting that gender is not merely a biological attribute but a socially constructed system that organizes power and opportunity in the workplace (West and Zimmerman, 1987). Within the Psychology of Working Theory (PWT), such gendered structures influence individuals’ access to decent work through contextual barriers, marginalization, and socialization processes (Blustein et al., 2016). Integrating gender theory into PWT highlights how systemic gender norms and labor market discrimination constrain vocational choice, work satisfaction, and well-being, particularly among women underscoring the need for equitable work policies and inclusive social frameworks.

## 2 Method

### 2.1 Participants and procedure

The data was collected between March and May 2024. The questionnaire was developed using Google Docs and distributed online through researchers’ contacts and various social media platforms to facilitate broad and rapid dissemination, allowing the survey to reach a diverse pool of potential respondents. The questionnaire had a first section with information about the purpose of the study and guarantee of confidentiality. After reading the informed consent form, the participants answered whether they were willing to participate; those who declined were directed to the end of the questionnaire. The participation was anonymous, and no identifying information was collected. All participants were informed about the study’s purpose, their right to withdraw at any time, and the confidential treatment of their responses. The research protocol was reviewed and approved by the Polytechnic of Coimbra Research Ethics Committee.

The final sample included 190 young adults, 57 men (30%) and 133 women (70%) aged 18 to 36 years old (M = 26.62; *SD* = 2.92). Most of them (77.4%) have a higher education. Participants worked in a variety of occupations in the public (72.1%) and private sector (27.9%) with 61.6% working from 35 to 40 h per week. Regarding schedules, 36% had fixed hours, 21% flexible, 19.5% in rotating shifts and 16.8% in shift schedule. Among men (*n* = 57), 16 held leadership position; among women (*n* = 133) only 8 did.

### 2.2 Measures

Decent work questionnaire by Ferraro et al. (2018) was used to assess young workers’ perceptions of decent work alongside with seven dimensions: (1) Fundamental Principles and Values (FPV); (2) Adequate Working Time and Workload (AWTW); (3) Fulfilling and Productive Work (FPW); (4) Meaningful Remuneration for the Exercise of Citizenship (MREC); (5) Social Protection (SP); (6) Opportunities (O); (7) Health and Safety (HS). The response scale ranged from 1 ‘completely disagree’ to 5 ‘completely agree’.

### 2.3 Data analysis

All the analyses were performed using version 28 of IBM SPSS Statistics. First, the mean scores of the variables under study were computed. The Cronbach’s alpha values for internal consistency ranged from 0.750 to 0.853, indicating good reliability (Pestana and Gageiro, 2008). Overall, participants scored higher in Health and Safety (HS) (*M* = 3.58, *SD* = 0.92) and Fundamental Principles and Values (FPV) (*M* = 3.55, *DP* = 0.83), while the lowest scores were observed in Social Protection (SP) (*M* = 2.46, *SD* = 0.96) and Meaningful Remuneration for the Exercise of Citizenship (MREC) (*M* = 2.74, *SD* = 0.93) (see Table 1).

**Table 1:** Descriptives of decent work dimensions including gender.

Decent work dimensions	Mean (SD)	Cronbach’s alpha reliability	Gender	
			Male M (SD)	Female M (SD)
Fundamental Principles and Values (FPV)	3.55 (0.83)	0.853	3.87 (0.61)	3.42 (0.87)
Adequate Working Time and Workload (AWTW)	3.16 (0.90)	0.801	3.20 (0.77)	3.15 (0.95)
Fulfilling and Productive Work (FPW)	3.46 (0.83)	0.750	3.68 (0.74)	3.37 (0.85)
Meaningful Remuneration for the Exercise of Citizenship (MREC)	2.74 (0.93)	0.814	3.12 (0.96)	2.58 (0.87)
Social Protect (SP)	2.46 (0.96)	0.827	2.72 (0.97)	2.34 (0.94)
Opportunities (O)	3.32 (1.02)	0.815	3.85 (0.85)	3.09 (1.01)
Health and Safety (HS)	3.58 (0.92)	0.829	3.72 (0.84)	3.52 (0.95)

Regarding gender differences, men reported higher scores than women across all the dimensions. Men scored highest in Fundamental Principles and Values (FPV) (*M* = 3.55, *DP* = 0.83), whilst women in Health and Safety (HS) (*M* = 3.52, *SD* = 0.9585). Both scored less in Social Protection (SP) (*M* = 2.72; *SD* = 2.34; *M* = 2.34, *SD* = 0.94, respectively).

### 2.4 Bivariate analysis

**Figure 1:**
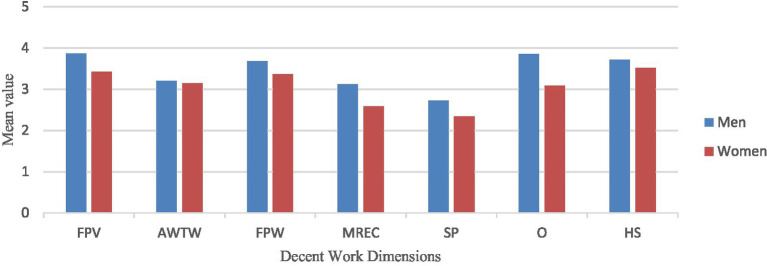
Means scores in decent work dimensions according to gender.

Significant differences in decent work dimensions according to gender are illustrated in Figure 1. Statistically significant differences were observed in most decent work variables based on gender, with men perceiving these variables more positively than women. A significant difference was found in FPV [*t*(149) = 4.09, *p* < 0.001, *d* = 0.56], with men reporting higher perceptions of Fundamental Principles and Values at Work (*M* = 3.87, *SD* = 0.61), compared to women (*M* = 3.42; *SD* = 0.87). Similarly, the results indicated a statistically significant difference in FPW scores between men and women [*t*(187) = 2.46, *p* = 0.007, *d* = 0.39], with men reporting perceiving Work as more Fulfilling and Productive (*M* = 3.68, *SD* = 0.74) than women (*M* = 3.37, *SD* = 0.85). Significant differences were observed in MREC [*t*(187) = 3.79, *p* < 0.001, *d* = 0.60], with men (*M* = 3.12, *SD* = 0.96) reporting higher perceptions of Meaningful Remuneration for the Exercise of Citizenship compared to women (*M* = 2.58, *SD* = 0.87). Likewise, significant differences were found in SP [*t*(187) = 2.49, *p* = 0.007, *d* = 0.39], with men (*M* = 2.72, *SD* = 0.97) perceiving greater Social Protection than women (*M* = 2.34, *SD* = 0.94). Additionally, a statistically significant difference was also found in (O) [*t*(187) = 5.30, *p* < 0.001, *d* = 0.78], with men (*M* = 3.85, *SD* = 0.85) perceiving more Opportunities at work than women (*M* = 3.09, *SD* = 1.01).

**Figure 2:**
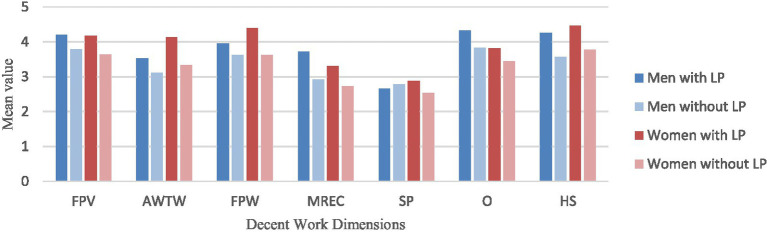
Means scores in decent work dimensions based on gender and leadership position. LP, Leadership position.

Gender differences were also assessed based on leadership role (see Figure 2) and work schedule, revealing statistically significant differences related to holding a leadership position. Men with leadership positions perceive to have more Fundamental Principles and Values at Work (FPV) [*t*(50) = −2,45, *p* = 0.018, *d* = −0.74], more Meaningful Retribution for the Exercise of Citizenship (MREC) [*t*(50) = −2.89, *p* = 0.006, *d* = −0.87], greater Opportunities (O) [*t*(50) = −2.60, *p* = 0.013, *d* = −0.78] and a better Health and Safety in the workplace (HS) [*t*(50) = −2.87, *p* = 0.006, *d* = −0.86], compared to those not holding leadership roles. Women in leadership positions, in turn, reported perceiving more Adequate Working Time and Workload (AWTW) [*t*(92) = −2.38, *p* = 0.02, *d* = −0.88], a more Fulfilling and Productive Work experience (FPW) [*t*(92) = −3.02, *p* = 0.003, *d* = −1.12] and better Health and Safety (HS) [*t*(92) = −2.18, *p* = 0.031, *d* = −0.81] conditions in the workplace, compared to those not in leadership roles. Significant differences were also observed with respect to work schedule type. Among women, the type of work schedule seems to influence the perception across all the decent work dimensions. In contrast, among men, a statistically significant difference was found only in the dimension Opportunities (O) [*F*(4,52) = 6.00, *p* = <0.001, *η^2^* = 0.32]. Men with fixed (*M* = 4.09, *SD* = 1.29), flexible (*M* = 4.09, *SD* = 0.70) and shift schedule (*M* = 3.68, *SD* = 0.73), reported perceiving more opportunities compared to the ones who have a rotating shift schedule (*M* = 2.45, *SD* = 1.29). Moreover, women with a flexible schedule, reported to experience a stronger presence of Fundamental Principles and Values at Work (FPV) [*F*(3,128) = 10.13, *p* < 0.001, *η^2^* = 0.19, *M* = 3.81, *SD* = 0.75], more Adequate Working Time and Workload (AWTW) [*F*(3,128) = 9.08, *p* < 0.001, *η^2^* = 0.18, *M* = 3.64, *SD* = 0.90], a more Fulfilling and Productive Work (FPW) [*F*(3,128) = 24.15, *p* < 0.001, *η^2^* = 0.29, *M* = 3.73, *SD* = 0.83] and better perceptions of Health and Security (HS) [*F*(3,128) = 10.19, *p* < 0.001, *η^2^* = 0.19, *M* = 3.90, *SD* = 0.97]. In turn, women with fixed work schedule perceive the Retribution for the Exercise of Citizenship as more meaningful (MREC) [*F*(3,128) = 6.47, *p* < 0.001, *η^2^* = 0.08, *M* = 2.75, *SD* = 0.95], higher Social Protection (SP) [*F*(3,128) = −9.69, *p* < 0.001, *η^2^* = 0.12, *M* = 2.61, *SD* = 0.94] and more Opportunities (O) [*F*(3,128) = 18.64, *p* < 0.001, *η^2^* = 0.30, *M* = 3.45, *SD* = 0.90]. On the other hand, having a rotating shift work schedule is considered by women as the work schedule type with a worst perception from women of decent work among all the study variables (see Table 2).

**Table 2:** Differences in decent work dimensions according to work schedule type.

Dimensions		FPVW		AWTW		FPW		MREC		SP		O		HS	
		M	F	M	F	M	F	M	F	M	F	M	F	M	F
Work Schedule Type	Fixed *M (SD)*	3.91 (0.54)	3.63 (0.88)	3.18 (0.91)	3.35 (0.95)	3.79 (0.70)	3.61 (0.82)	3.08 (1.12)	2.75 (0.95)	2.66 (0.93)	2.61 (0.94)	4.09 (0.70)	3.45 (0.90)	3.91 (0.90)	3.79 (0.90)
	Flexible *M (SD)*	4.09 (0.41)	3.81 (0.75)	3.59 (0.60)	3.64 (0.90)	3.80 (0.77)	3.73 (0.83)	3.27 (0.92)	2.69 (0.93)	2.70 (1.08)	2.50 (1.09)	4.08 (0.58)	3.45 (0.87)	3.81 (0.73)	3.90 (0.97)
	Shifts *M (SD)*	3.72 (0.77)	3.29 (0.84)	3.00 (0.52)	2.89 (0.84)	3.55 (0.68)	3.51 (0.50)	3.10 (0.86)	2.62 (0.91)	2.92 (0.71)	2.29 (0.91)	3.65 (0.73)	3.21 (0.73)	3.54 (0.76)	3.38 (0.96)
	Rotating *M (SD)*	3.43 (0.69)	2.80 (0.62)	2.70 (0.78)	2.55 (0.72)	3.24 (0.95)	2.58 (0.54)	2.55 (0.62)	2.17 (0.44)	2.45 (1.23)	1.80 (0.54)	2.45 (1.29)	2.13 (0.83)	3.10 (0.63)	2.84 (0.62)
Test	*F*	1.47	10.13	2.03	9.08	0.74	24.15	0.780	6.47	0.243	9.69	6.00	18.19	1.15	10.19
	*p*	0.23	**<0.001**	0.104	**<0.001**	0.567	**<0.001**	0.543	**<0.001**	0.913	**<0.001**	**<0.001**	**<0.001**	0.35	**<0.001**

M, mean; SD, standard deviation; M, male; F, female. Bold values indicate statistically significant differences.

## 3 Discussion

The present findings offer important insights into young workers’ perceptions of decent work conditions across several key dimensions. Notably, the highest average scores were recorded for Fundamental Principles and Values (FPV) and Health and Safety (HS), suggesting that young workers generally felt the presence of values such as justice and respect and are somehow satisfied with workplace safety conditions. However, it should be noted that the moderate score for Opportunities (M = 3.32, SD = 1.02) may reflect their perceptions of limited access to professional growth and career advancement, while the moderate score of Health and Safety (M = 3.58, SD = 0.92) is likely to indicate that a baseline level of physical and psychological safety is perceived to be assured but there is room for improvement of this dimension. Social Protection (SP) and Meaningful Remuneration for the Exercise of Citizenship (MREC) were rated lowest among all dimensions. The low mean score for Social Protection (M = 2.46, SD = 0.96) raises concerns about the adequacy of benefits, security, and social welfare provisions for young workers. Similarly, the limited sense of MREC (M = 2.74, SD = 0.93) implies that young workers may feel salaries are low and do not allow them to have good living conditions. When gendered differences are considered, a consistent pattern emerged: men reported higher scores across all dimensions of decent work compared to women. This disparity may reflect structural and cultural differences in how work is experienced by gender. For example, while both men and women identified Health and Protection as one of the most positive aspect of their work lives, men scored this dimension substantially higher (M = 3.72, SD = 0.84) than women (M = 3.09, SD = 1.01). Similarly, although Social Protection was the lowest-rated dimension for both genders, the gender gap persisted (men: M = 2.72, SD = 2.34; women: M = 2.34, SD = 0.94), suggesting that women may face greater insecurity or perceive fewer institutional protections in their work environments. These findings are consistent with international evidence highlighting the gendered nature of labor experiences. Across many countries, men tend to enjoy greater opportunities for upward mobility and stronger institutional protections, whereas women frequently face structural barriers that restrict their full participation and recognition in the workforce (OECD, 2021). The low ratings for MREC further reinforce concerns about the gender disparities in salaries. The findings provide valuable insight into how young workers perceive various dimensions of decent work and the persistence of gender differences in this analysis revealing some critical gender gaps. While young workers scored highest in Health and Safety (HS) and Fundamental Principles and Values (FPV), suggesting that for many, work expands and promotes an active participation in values such as respect, justice, and even mental health and is conducted in relatively secure and healthy environments, these results echo the central aspects of Blustein’s Psychology of Working Theory (PWT) (Blustein et al., 2016; Duffy et al., 2016). However, the notably lower scores in Social Protection (SP) and Meaningful Remuneration for the Exercise of Citizenship (MREC) points to significant shortcomings in young workers’ experiences. The gender disparities observed further illustrate structural inequities in access to decent work. Across all dimensions, men reported higher scores than women. This pattern aligns with PWT’s emphasis on the role of contextual and systemic factors—such as gender norms and labor market discrimination—in shaping access to decent work. Women’s lower scores in SP and MREC are especially concerning, as they indicate heightened vulnerability in the workplace (Ferreira and Coelho, 2022; OECD, 2021). These disparities may reflect broader societal patterns in which women’s labor is undervalued or unsupported by institutional frameworks.

In sum, while some aspects of decent work are being met, particularly related to the presence of important values and safety, the findings reveal key deficits in social protection and recognition, especially for women. From a PWT perspective, this indicates that many young workers are not fully achieving the psychological and material fulfillment that decent work should offer. Addressing these gaps requires intentional policy interventions and workplace practices that foreground equity, empowerment, and structural inclusion.

Results indicated that holding a leadership position influences the perception of decent work, with gender-specific trends. Men in leadership roles reported more positive perceptions of FPV, MREC, Opportunities, and Health and Safety than their counterparts in non-leadership roles. This suggests that leadership roles may offer a protective or empowering effect, potentially providing men with greater autonomy and recognition in their workplaces. Interestingly, women in leadership positions also reported significantly better experiences, particularly in terms of Working Time and Workload (AWTW), Fulfilling and Productive Work experience (FPW), and Health and Safety (HS). These findings point to the potential benefits of leadership roles for women’s work satisfaction. However, the effect sizes were especially strong among women, such as in FPW (*d* = −1.12), suggesting that occupying leadership positions may be particularly impactful for women in reshaping otherwise less favorable perceptions of work.

The analysis of the gender differences considering the work schedule type emerged also as a critical factor in shaping women’s experiences of decent work. Women with flexible schedules reported significantly more positive perceptions across nearly all dimensions, including FPV, AWTW, FPW, and HS. Women with fixed schedules reported greater MREC, SP, and Opportunities. In contrast, women with rotating shifts reported the lowest levels of decent work perceptions, suggesting that irregular schedules may exacerbate negative work experiences for women. For men work schedule type had a far less pronounced impact. The only statistically significant finding among male participants was that those with fixed, flexible or shift schedules perceived greater Opportunities than those with rotating shifts. This divergence suggests that, in line with previous research that women are more vulnerable to the structural effects of scheduling practices, likely due to the intersection of work responsibilities and external demands that disproportionately affect them (Ferreira and Coelho, 2022; OECD, 2021).

## 4 Conclusion

Overall, the findings open up interesting avenues for exploring gender inequalities in the perceptions of decent work. The patterns observed suggest that gender may shape how individuals experience and evaluate different dimensions of decent work, pointing to the need for further research that examines these dynamics more closely. Future studies could investigate how structural, cultural, and occupational factors interact with gender to influence these perceptions, thereby contributing to a deeper understanding of gendered experiences in the workplace. In fact, this study highlights how gender shapes individuals’ perceptions and experiences of decent work by revealing gender-specific patterns in how young adults evaluate various dimensions of decent work. With this approach, the study contributes to a relatively underexplored area in the literature on how gender inequalities manifest, not only in objective working conditions but also in subjective understandings of what constitutes decent work. A limitation of this study that should be noted is the gender imbalance within the sample which might affect the generalizability of the findings. Because women are disproportionately represented, the results might not fully capture potential differences or similarities across genders. It should also be noted that the reliance on voluntary online participation introduces self-selection bias, potentially excluding young people in more precarious working or health conditions (e.g., burnout), who are less likely to engage in such surveys despite being central to the phenomenon under study. Future research should aim to replicate these findings using more gender-balanced and diverse samples to ensure that the conclusions drawn are robust and applicable to a broader population.

## 5 Implications

These results underscore the need for gender-responsive workplace policies. The consistent gender gap in perceptions of decent work points to deeply rooted inequities in the labor environment (International Labour Organization, 2022). Leadership opportunities and flexible scheduling appear to buffer some of these effects, especially for women, highlighting critical ways for intervention. Efforts to promote decent work must address the structural conditions that reproduce gender disparities, including the design of work schedules, access to leadership, and inclusive organizational cultures (International Labour Organization, 2022). Policies that support flexible work, equitable career progression, and family-friendly environments could play a vital role in reducing these disparities and fostering workplaces that are genuinely inclusive (OECD, 2021).

From the educational perspective early interventions through career education can also play an important role for young adults entering the labor market (OECD, 2021). Career guidance programs should address gender stereotypes and inform young women of their rights, entitlements, and available opportunities to empowering young workers, especially young women, with this knowledge enables them to make informed decisions and better navigate structural challenges in the workforce.

## Data Availability

The original contributions presented in the study are included in the article/supplementary material, further inquiries can be directed to the corresponding author.
